# The Influence of Genetic Variability and Proinflammatory Status on the Development of Bone Disease in Patients with Gaucher Disease

**DOI:** 10.1371/journal.pone.0126153

**Published:** 2015-05-15

**Authors:** Javier Gervas-Arruga, Jorge Javier Cebolla, Ignacio de Blas, Mercedes Roca, Miguel Pocovi, Pilar Giraldo

**Affiliations:** 1 Centro de Investigación Biomédica en Red de Enfermedades Raras (CIBERER), Zaragoza, Spain; 2 Translational Research Unit, Miguel Servet University Hospital, Zaragoza, Spain; 3 Spanish Gaucher Disease Foundation (FEETEG), Zaragoza, Spain; 4 Instituto Aragonés de Ciencias de la Salud (IACS), Zaragoza, Spain; 5 Departamento de Bioquímica, Biología Molecular y Celular, Universidad de Zaragoza, Zaragoza, Spain; 6 Unidad de Patología Infecciosa y Epidemiología, Facultad de Veterinaria, Universidad de Zaragoza, Zaragoza, Spain; 7 International Skeletal Society-Radiodiagnostic Center, Zaragoza, Spain; University Hospital S. Maria della Misericordia, ITALY

## Abstract

Gaucher disease, the most common lysosomal storage disorder, is caused by β-glucocerebrosidase deficiency. Bone complications are the major cause of morbidity in patients with type 1 Gaucher disease (GD1). Genetic components strongly influence bone remodelling. In addition, chronic inflammation produced by Gaucher cells induces the production of several cytokines, which leads to direct changes in the bone remodelling process and can also affect the process indirectly through other immune cells. In this study, we analysed the association between bone mineral density (BMD), bone marrow burden score, and relevant genetic polymorphisms related to bone metabolism, as well as profiles of proinflammatory cytokines in a GD1 cohort. This study included 83 patients distributed according to bone status. BMD was measured with DXA and broadband ultrasound attenuation; bone marrow involvement was evaluated using MRI. We also analysed 26 SNPs located in 14 genes related to bone metabolism. To assess proinflammatory status, we analysed IL-4, IL-6, IL-7, IL-10, IL-13, MIP-1α, MIP-1β, and TNFα in plasma samples from 71 control participants and GD1 patients. SNP genotype proportions and BMD differed significantly between *ESRI* c.453-397T>C and *VDR* c.1024+283G>A variants. We also observed significant associations between GD1 genotypes and bone affectation. When patients were stratified by spleen status, we observed significant correlations between non-/splenectomized groups and Spanish MRI (S-MRI) score. Across genotype proportions of non-/splenectomized patients and S-MRI, we observed significant differences in *ESRI* c.453-397T>C, *VDR* c.-83-25988G>A, and *TNFRSF11B* c.9C>G polymorphisms. We observed different significant proinflammatory profiles between control participants, treatment-naïve patients, and patients on enzyme replacement therapy (ERT); between non-/splenectomized patients (between untreated and ERT-treated patients) and among those with differing *GBA* genotypes. The data suggest that patients with GD1 have increased susceptibility to developing bone disease owing to the coexistence of genetic variants, and that genetic background in GD1 is fundamental to regulate the impact of proinflammatory status on the development of bone disease.

## Introduction

Gaucher disease (GD) (OMIM#230800), the most common lysosomal disease, is an autosomal recessive disorder caused by a deficiency of the enzyme β-glucocerebrosidase (EC 3.2.1.45). This disease is characterized by spleen and liver enlargement, cytopenias, and bone marrow infiltration[1]. Bone complications are a major cause of morbidity and one of the most debilitating aspects of type 1 GD (GD1). More than 80% of GD1 patients have bone involvement [2]. The progressive storage of glucocerebroside in the bone marrow is associated with osteopenia and osteoporosis, which may to lead fractures, avascular bone necrosis, cortical thinning, lytic bone lesions, osteosclerosis, and (rarely) acute osteomyelitis [3].

In adults, bones are continually renewed through a process known as “bone remodeling”. The old bone areas are removed by osteoclasts and replaced by new bone tissue formed by osteoblasts. Bone remodeling takes place throughout the entire skeleton, and it has been calculated that approximately 20% of trabecular bones and 10% of compact bones are involved in this process [4]. The most important factors that influence bone remodeling are genetic components, which may explain between 65% and 90% of bone mass variability [5].

Today, a rather high number of candidate genes that regulate bone mineral density (BMD) and enhance susceptibility to osteoporosis have been identified [6]. Most genes have been selected based on their role in regulating calcium metabolism or the function of calcium in bone cells. However, in many cases, the precise underlying mechanism associating these genes with BMD is unknown. In addition, in GD1, bones may be affected by several complex pathological mechanisms. The central hypothesis is based on Gaucher cell infiltration, which alters vascularity and increases local pressure owing to extensive glucocerebroside accumulation. Gaucher cells do not directly induce bone resorption. Chronic inflammation produced by Gaucher cells induces the production of several cytokines, which can lead to changes in the bone remodeling process directly, or can act on it indirectly through various other cells of the immune system [7].

Changes in levels of cytokines such as IL-6 and TNF-α influence bone remodeling cells that appear to be relevant to the development of osteopenia in GD1. Furthermore, the macrophage inflammatory proteins (MIPs) MIP-1α and MIP-1β, which have been shown to increase bone resorption by osteoclasts in multiple myeloma [8], were also elevated in GD with bone disease [9, 10]; therefore, in combination with other cytokines, MIP-1α and MIP-1β might also contribute to pathological skeletal alterations in GD1. Besides the role of altered macrophage function on bone turnover mediated by proinflammatory interleukins, we cannot exclude a possible role of other cells implicated in bone remodeling process such as osteoblasts as shown by Mistry et al [11] in glucocerobrosidase gene-deficient mouse.

Before enzyme replacement therapy (ERT) became available, splenectomy was the only method that improved disease status in patients affected with severe cytopenias, functional hypersplenism, or local mechanical pressure caused by extensive splenomegaly. However, clinical findings have demonstrated that, over time, splenectomy negatively affects the course of bone disease in GD. Splenectomized GD patients had higher bone marrow scores indicative of severe bone disease than non-splenectomized patients [12]. Splenectomized GD patients also experienced bone manifestations that were more progressive over time compared with non-splenectomized GD patients [12].

In the study reported here, we analyzed the association between BMD, the bone marrow burden Spanish MRI (S-MRI) score, and relevant genetic polymorphisms related to bone metabolism in a cohort of GD1 patients. We also assessed the profiles of proinflammatory cytokines related to the development of bone disease.

## Materials and Methods

### 2.1-Study population

A retrospective, analytical study was performed using frozen DNA and plasma samples (stored at -80°C) from Spanish GD1 patients diagnosed between 1995 and 2004; patients were followed for at least 6 years, and their clinical, analytical, and image data was recorded in the Spanish Gaucher Disease Registry (SGDR] [13]. The SGDR is authorized in accordance with the rules of the Aragon Ethical Committee (CEICA). For this study, 83 patients were selected and distributed according *GBA* genotype and presence/absence of bone disease. Patients with monoclonal gammopathy, multiple myeloma, or another associated neoplasia were excluded. A group of 71 healthy control subjects, without bone disease, was subjected to cytokine analysis. To compare ethnicity-based genetic differences between our population and the European population, we used the 1000 Genomes database (www.1000genomes.org). Written informed consent was obtained from all patients including those from the parents on the behalf of the minors involved in our study. The study was approved by the Ethics Committee of Aragon (CEICA) and was conducted in accordance with the Helsinki declaration of 1975, as revised in 2000.

### 2.2-Anthropometric data

Body Mass Index (BMI) was computed as weight divided by squared height (kg/cm^2^). Patients were classified according to BMI score as underweight (<18.5), normal (18.5–24.99), overweight (25–29.99), or obese (≥30). Ten patients had no recorded weight or height. We used Spanish anthropometric standards to classify patients younger than 18 years of age [14].

### 2.3-Bone disease assessment

Each individual underwent a full clinical, analytical, and image evaluation before therapy was initiated. BMD was measured at the femoral neck and L 1–4 using DXA (LUNAR, GE Medical Sytems) and at the calcaneus using broadband ultrasound attenuation (BUA; Norland, CUBA clinical). Ultrasound was completed by the same observer and under the same conditions for all patients. We used World Health Organisation criteria to classify patients as normal, osteopenic, or osteoporotic based on the normative values for young patients [15]. We used Z-score for patients <50 years old and T-score for patients ≥50 years old. We considered a patient to have osteoporosis when patients showed a significant decrease of BMD compared to population of same sex and age. Bone marrow involvement was evaluated through serial MRI of the spine, pelvis, and femurs. Spin echo; T1 and T2-weighted sequences were performed. S-MRI scores were calculated according to MRI infiltration patterns [16], and patients were classified as Low-Normal (0–5), Mild (6–10), or Severe (>10).

### 2.4-DNA analysis

Genomic DNA was isolated from whole blood using standard procedures. To study patients’ genetic background, we analyzed 26 single-nucleotide polymorphisms (SNPs) located in 14 genes related to bone metabolism (Table A in S1 File).

### 2.5-Genotyping

We used restriction fragment length polymorphism (RFLP) analysis to assess the following polymorphisms: c.104-441G>T and c.-2116T>G SNPs of *COL1A1*; c.152T>A, c.1024+283G>A, c.1025-49G>T, c.1056T>C, c.-83-23269A>G, and c.-83-23777G>C of *VDR*; c.453-397T>C of *ESR1*; c.-208G>A and c.1073A>C of *IL6R*; c.1180G>A of *CLCN7*; c.455T>C of *BMP4*; and c.-223C>T of *TNFRSF11B*. The microsatellite (TA)n repeat in the promoter region of *ESR1* was analysed by capillary electrophoresis and sequencing was used to type the c.-1782delT of *COL1A1* and the c.-260_-259insG of *OPN*. The SNaPshot method (Applied Biosystems) was used to type the following polymorphisms: c.453-351A>G of *ESR1*, c.-83-25988G>A of *VDR*, c.59-1041T>C of *RUNX2*, c.219+2528T**>C** of *TNFSF11*, c.142+76A>G of *TGFB1*; c.9C>G of *TNFRSF11B*, c.196G>A of *BDNF*, c.-48-7324A>T of *HSD11B1* and c.-94C>G *VEGF*. Further details on the methods are provided in Text A in S1 File.

### 2.6-Cytokine assay

We analysed 25-μl plasma samples from patients for eight cytokines in duplicate (IL-4, IL-6, IL-7, IL-10, IL-13, MIP-1α, MIP-1β, and TNFα) using the Human Cytokine Lincoplex Kit (MPXHCYTO-60K-23; Millipore, Linco Research Inc.). Assays were performed according to the manufacturer’s recommendations. A standard curve covering the 3.2–10.000 pg/mL concentration range was generated by serially diluting reconstituted standards. Fluorescence measures were acquired using the Luminex100 platform (Luminex Corporation). Data were collected and analysed with Luminex xPONENT software (Luminex Corporation). A five-parameter regression formula was used to calculate sample concentration from the standard curves.

### 2.7-Statistical methods

Statistical analyses were performed using the Statistical Package for the Social Sciences software, version 20.0 (IBM SPSS Inc., Chicago, IL). The chi-squared test was used to compare proportions across groups; associations between individual SNPs, BMD, and spleen status; and allele and genotype frequencies for each SNP. Effect sizes were evaluated using a likelihood function. We tested Hardy-Weinberg Equilibrium (HWE) by comparing the observed genotype with the expected genotype frequency using the chi-square test. Variable distribution normality across cytokine groups was analysed using the Kolmogorov-Smirnov test, and mean or median comparison was performed by a parametric one-way ANOVA using Bonferroni post hoc or non-parametric Mann-Whitney U and Kruskall-Wallis tests.

## Results

### 3.1-Cohort study

**Fig 1:**
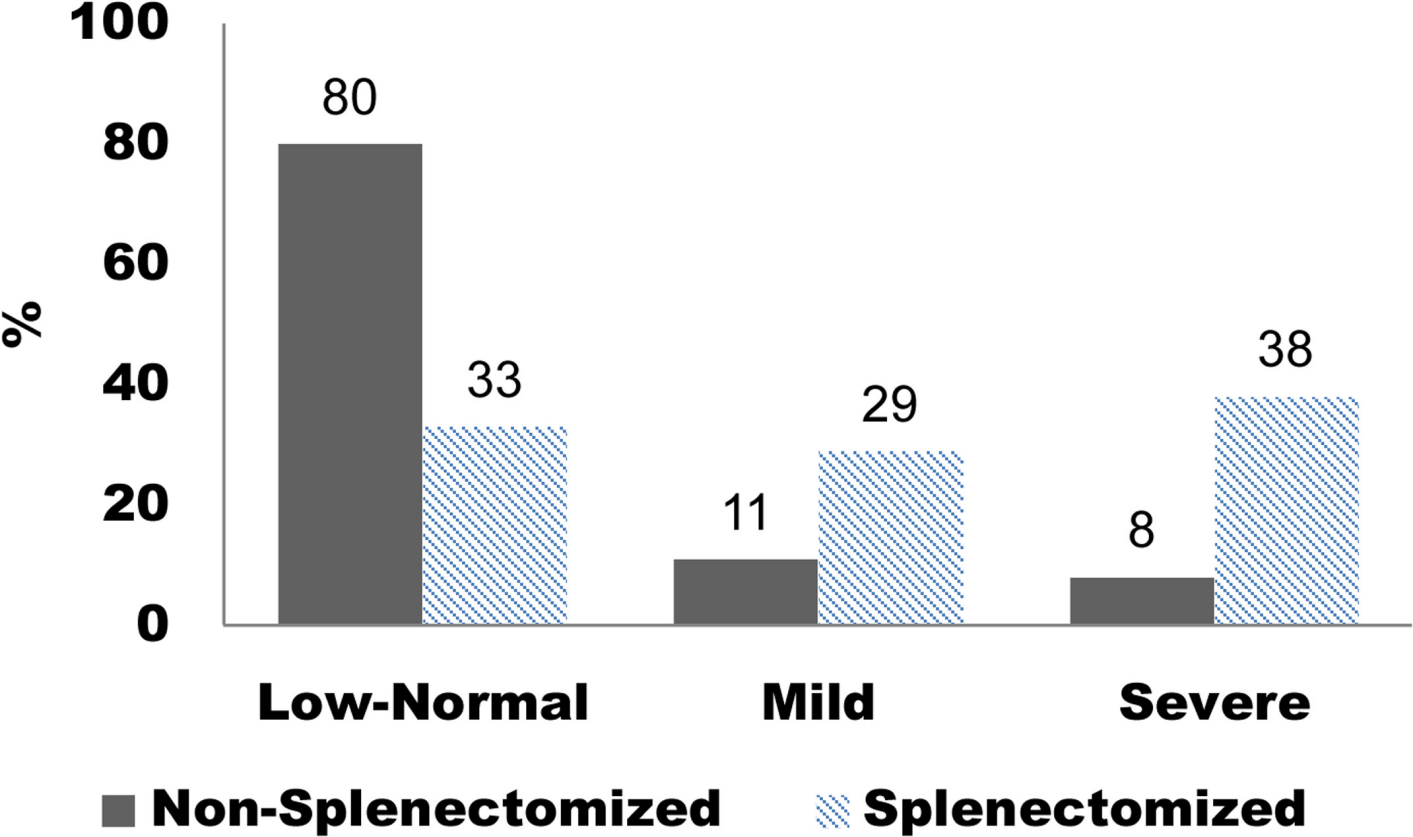
S-MRI scores among non-splenectomized versus splenectomized GD1 patients (*p* = 0.0001). Low-normal (0–5), Mild (6–10), Severe (>10).

The clinical data obtained from clinical records and bone characteristics of 83 GD1 patients are detailed in Table 1. BMD was measured in 77 patients and BUA was calculated for 44 patients. We found significant differences in median BUA values between normal and osteopenic patients (Fig A in S1 File). We also observed significant associations between GD1 genotypes and BMD (*p* = 0.004; Table 2). BMD and S-MRI score were not related to BMI or gender (data not shown). When stratified by spleen status, the patients demonstrated significant correlations between non-/splenectomized groups and S-MRI (*p* = 0.001) (Fig 1). To analyse the proinflammatory cytokine profile, we analyze GD1 plasma samples from members of this cohort treatment-naïve (n = 46/83 (55.4%), mean age 39±20 years); during ERT (n = 42/83 (50.6%), median age 42±16, imiglucerase therapy 3–6 years, 15–60 U/kg every other week) and control plasma samples (n = 71, mean age 53±18 years).

**Table 1:** General and Clinical Cohort Details.

**Sex, n (%)**				
Male	43 (51.8)			
Female	40 (48.2)			
**Age, years** ^**a**^	40.5±18 (5–79)			
**Genotype,**	N370S/N370S	N370S/L444P	N370S/Others	
**n (%)**	14 (16.8)	48 (57.9)	21 (25.3)	
**Spleen Status,**	Non Splenectomized	Splenectomized		
**n (%)**	62 (74.7)	21 (25.3)		
**Clinical history bone impairment,**	Bone pain	Avascular necrosis/infarcts	Orthopaedic procedures	
**n (%)**	35 (41.2)	33 (38.8)	12 (14.5)	
**BMI,**	Normal	Underweight	Overweight	Obese
**n (%)** ^**b**^	52 (71.2)	5 (6.8)	12 (16.4)	4 (5.5)
**DXA, n (%)**	29 (37.6)			
**BMD,**	Normal	Osteopenia	Osteoporosis	
**n (%)** ^**c**^	14 (48.3)	10 (34.5)	5 (17.2)	
**US, n (%)**	48 (62.4)			
**BMD,**	Normal	Osteopenia	Osteoporosis	
**n (%)** ^**c**^	29 (60.4)	15 (31.3)	4 (8.3)	
**BUA(n),**	Normal(25)	Osteopenia (15)	Osteoporosis(4)	
**db/Mhz** ^**a**^	82.36±14.07	55.43±11.43	38.37±8.6	
**S-MRI,**	Low-Normal	Mild	Severe	
**n (%)** ^**d**^	57 (68.7)	13 (15.7)	13 (15.7)	

**a** Values presented as mean±SD (range)

**b** Adjusted for age

**c** World Health Organization criteria classification Z-score, T-score adjusted for age

**d** S-MRI score classification: Low-Normal (0–5), Mild (6–10), Severe (>10).

**Table 2:** GD1 Genotype and BMD^a^.

GD1 Genotype	Bone Affectation		*p* = 0.004
	Normal, n (%)	Affected, n (%)^b^	n
N370S/N370S	9 (75)	3 (25)	12
N370S/L444P	28 (66.6)	14 (33.3)	42
N370S/others	7 (30.4)	16 (69.5)	23

a Measured by DXA and US

b Osteopenic and osteoporotic patients

### 3.2-Genetic screening

The allele frequencies of polymorphisms are described in Table A in S1 File. All of the genotype distributions were in Hardy-Weinberg equilibrium (*p*<0.05) except for the following SNPs: *RUNX2* c.59-1041T>C, *BDNF* c.196G>A, *VDR* c.-83-23269A>G, and *COL1A1* c.-2116T>G (Table A in S1 File). A significant difference in allelic frequencies between this group of Spanish GD patients and the European population reported in the 1000 Genomes database were observed in: *HSD11B1* c.-48-7324A>T; *ESRI* c.453-397T>C and c.453-351A>G; *TNFSF11* c.219+2528T>C; *BDNF* c.196G>A; *VDR* c.1025-49G>T; *COL1A1* c.-2116T>G; *IL6R* c.-208G>A; and *BMP4* c.455T>C (Table A in S1 File). Moreover, we observed significant associations among GD1 genotypes and the following SNPs: *VDR* c.-83-23269A>G (*p* = 0.027) and c.-83-23777G>C (*p* = 0.007); *BMP4* c.455T>C (*p* = 0.014); *RUNX2* c.59-1041T>C (*p* = 0.01); and *TGFβ1* c.142+76A>G (*p* = 0.038). We also observed significant differences between SNP genotype proportions and BMD in *ESRI* c.453-397T>C (*p* = 0.038) and *VDR* c.1024+283G>A variants (*p* = 0.039) (Table B in S1 File). We found significant differences across genotype proportions of splenectomized patients and S-MRI in *ESRI* c.453-397T>C (*p* = 0.047) and *VDR* c.-83-25988G>A (*p* = 0.045) polymorphisms, as well as for non-splenectomized patients and S-MRI in *TNFRSF11B* c.9C>G (*p* = 0.040) (Table C in S1 File).

### 3.3-Cytokine screening

**Fig 2:**
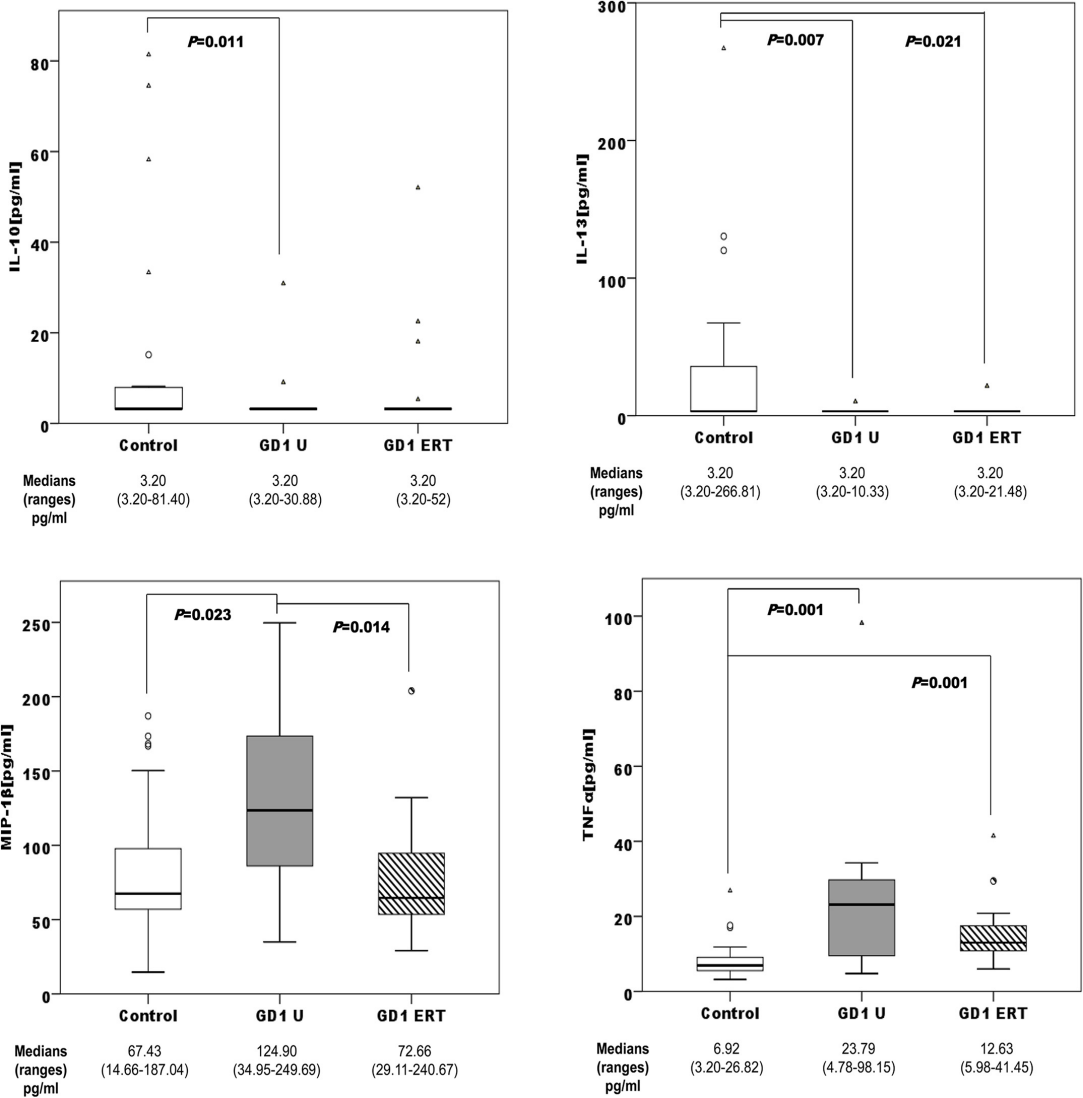
Distribution of IL-10, IL-13, MIP-1β, and TNFα concentrations among male controls (n = 25); male treatment-naïve GD1 patients (GD U; n = 21), and male ERT-treated GD1 patients (GD1ERT; n = 18). ERT: Imiglucerase, 15–60 U/kg every other week for 3–6 years.

When we stratified the series by sex, we found a significant profile among control group, treatment-naïve, and ERT-treated patients. Among males, the untreated patients presented altered levels of IL-10, IL-13, MIP-1β, and TNFα compared with the male control group. The male patients on ERT presented altered levels of IL-13, and TNFα compared with the male control group. Finally, we observed significant differences in MIP-1β levels between the untreated male patients and the ERT-treated male group (Fig 2).

**Fig 3:**
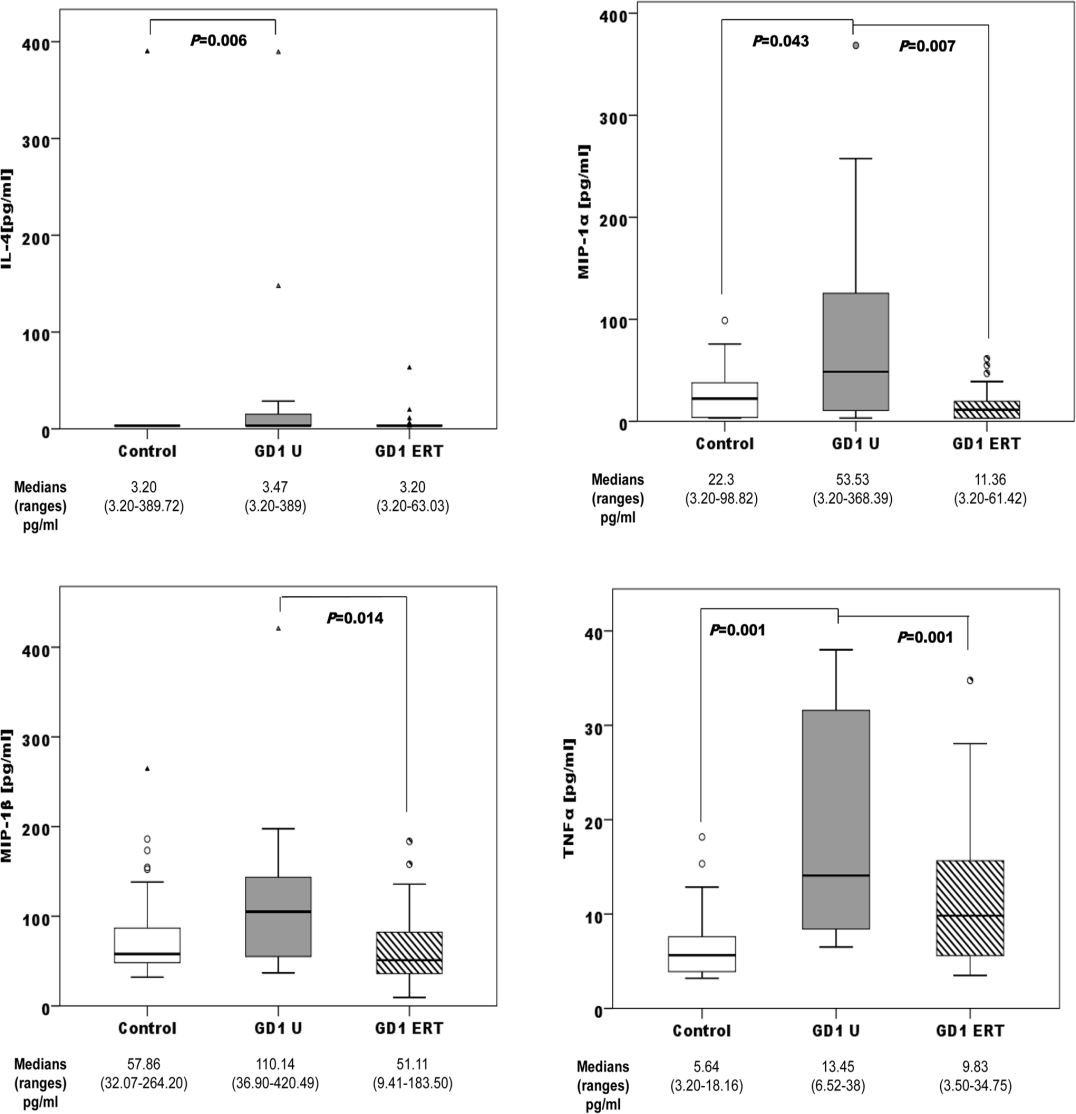
Distribution of IL-4, MIP-1α, MIP-1β, and TNFα concentrations among female controls (n = 46); female treatment-naïve GD1 patients (GD U; n = 25), and female ERT-treated GD1 patients (GD1ERT; n = 24). ERT: Imiglucerase, 15–60 U/kg every other week for 3–6 years.

Treatment-naïve female patients presented altered levels of IL-4, MIP-1α, and TNFα compared with females of the control group. The female patients on ERT presented altered levels of TNFα compared with the female control group. We also observed significant differences in MIP-1α and MIP-1β levels between untreated female patients and ERT-treated females (Fig 3).

**Fig 4:**
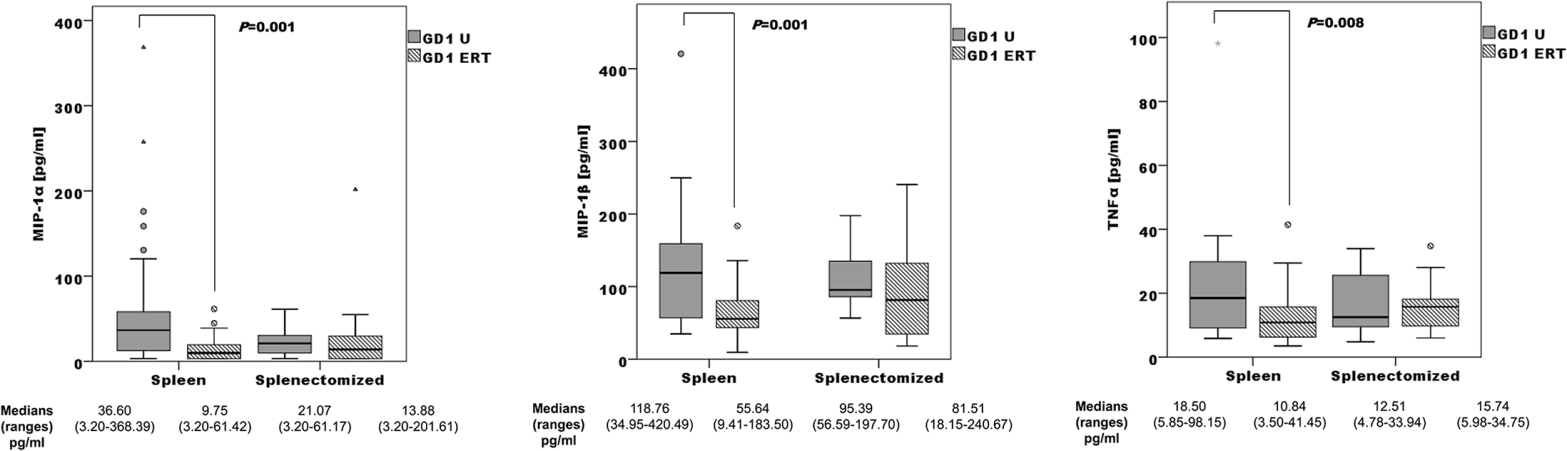
Comparison of the distribution of MIP-1α, MIP-1β, and TNFα concentrations: treatment-naïve (GD1U; n = 40) versus ERT-treated (GD1ERT; n = 27) non-splenectomized patients, and treatment-naïve (GD1U; n = 6) versus ERT-treated (GD1ERT; n = 15) splenectomized patients.

Surprisingly, we did not observe significantly different cytokine profiles according to BMD or S-MRI score. We observed a non-significant profile difference between non-/splenectomized patients, in both untreated and ERT-treated patient groups (Fig B in S1 File). In non-splenectomized patients, levels of MIP-1α, MIP-1β, and TNFα differed significantly between GD1 untreated and GD1 ERT-treated patients. These differences are not evident in the splenectomized group and GD1 ERT-treated (Fig 4).

### 3.4-Relationship between genetics and plasma proinflammatory profiles

**Fig 5:**
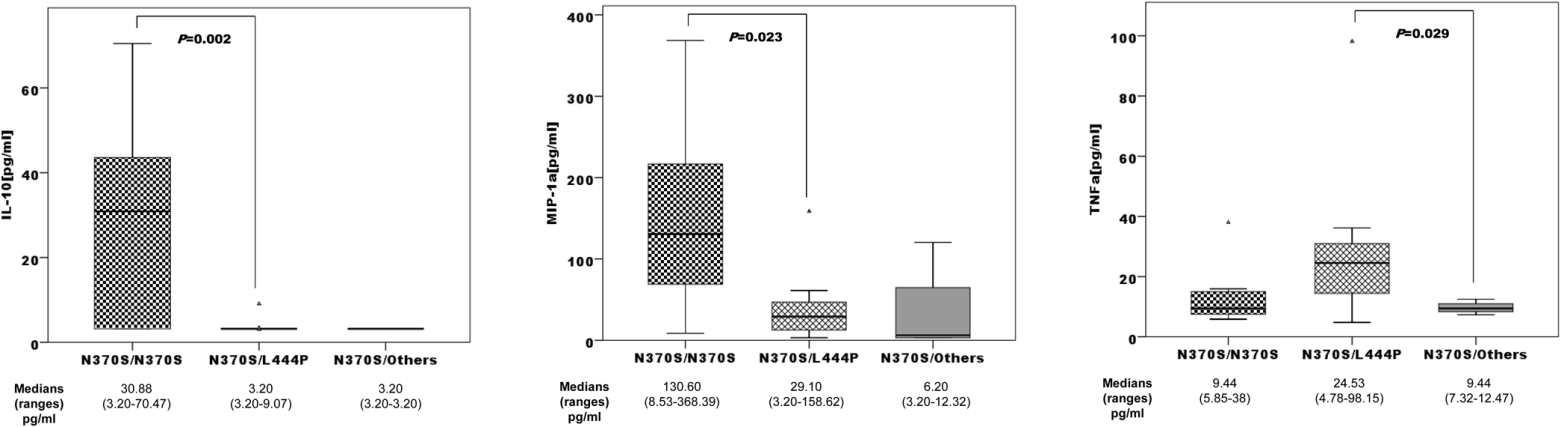
Distribution of IL-10, MIP-1α, and TNFα concentrations among GD1 genotypes N370S homozygous (n = 9); N370S/L444P (n = 31), N370S/others (n = 6).

In plasma samples from untreated patients, levels of IL-10, MIP-1α, and TNFα differed significantly with respect to GD1 genotype (Fig 5). However, we observed significant differences among SNP genotypes and different plasma cytokine concentrations (Table D in S1 File). Patients’ BUA (db/MHz) revealed significant differences between genotypes of SNP *VDR* c.-83-23269A>G (A/A = 79; G/A = 64; G/G = 82, (*p* = 0.036)).

## Discussion

Several studies suggest that bone turnover biomarkers may be disturbed in GD [17]. BMD is an important clinical predictor of fracture risk [18] but a clear relationship between a low bone mass and risk of fractures is not yet well established. The absolute risk of osteoporotic fractures increases significantly with age at the same level of bone mass. Both age and prior fracture are strong predictors of future fractures [19]. We evaluated BMD using DXA in 37.6% of cases and calcaneus ultrasound in 62.4% of cases. Several studies have shown that BUA and DXA have the same predictive value [20, 21]. The hypothesis that gene variability may be a predictive value of osteoporosis risk is supported by the importance of genetic background in the regulation of bone metabolism. To study the genetic background of the study participants, we analyzed 26 SNPs located in 14 genes related to bone metabolism that had been associated with BMD loss in previous studies [5, 22–25]. Genotypic frequencies were in Hardy-Weinberg equilibrium, except for *RUNX2* c.59-1041T>C, *BDNF* c.196G>A, *VDR* c.-83-23269A>G, and *COL1A1* c.-2116T>G. A small but significant difference was observed, which suggests that in these four polymorphisms our population not was in genetic equilibrium owing to a small sample size effect.

Some ethnic differences have been demonstrated in relation to other populations. Results of our comparison with the 1000 Genomes database demonstrated that allele and genotype frequencies in this GD cohort were similar to those of the European population. We found significant differences between our cohort and the 1000 Genomes database in 8 of 26 SNPs (Table A in S1 File). We also found a significant genetic protective profile against BMD loss and bone infiltration or bone lesions. In this sense, the genotypes *ESRI* c.453-397 T/T and *VDR* c.1024+283G A/A each demonstrated a significant association with protective BMD loss (Table B in File S1). These results are consistent with other previously published results. *VDR* SNP c.1024+283G>A (known as BsmI) has been correlated with BMD, particularly with respect to Z-score and skeletal involvement in GD [26], and c.1024+283GG + c.1024+283 GA genotypes are more frequent in patients with osteoporotic fractures [24]. The c.1024+283G>A SNP is located in the 3’ untranslated region of the vitamin D receptor gene (*VDR*). It is a steroid receptor that acts as a transcription factor in response to the active form of vitamin D hormone. This hormone plays an important role in skeletal metabolism, including intestinal calcium absorption and the regulation of osteoblast differentiation [27]. SNP *VDR* c.-83-23269A>G differed significantly with respect to mean BUA (db/MHz) value and genotype. Mean BUA values are significantly lower among osteopenic patients than among normal GD1 patients (Figure A in S1 File). A genome-wide association study (GWAS) reported that five SNPs in the *ESR1* gene exhibited an association with BMD of both the hip and spine, suggesting a possible role for the *ESR1* gene in the pathogenesis of osteoporosis [22]. A more recent GWAS further confirmed the association between the *ESR1* gene and osteoporotic fractures [28]. The most studied variants of the *ESR1* gene are the c.453-397T>C and c.453-351A>G (known as XbaI) polymorphisms, which have been linked to reduced estrogen sensitivity [29]. The estrogen *ESR1* complex is primarily responsible for regulating cellular signaling pathways in vivo, as well as bone mass in skeletal systems [30]. Serum estradiol level may be a predictor of subsequent BMD [31] and risk for osteoporotic fractures [32]. Although *GBA*1 genotype is not a critical factor for low BMD [33], patients with hetero-allelic N370S tend to have more hematological and visceral manifestations in GD1 [34]. We found associations between *GBA1* genotypes, different genetic bone metabolism SNPs, and bone affectation; 69.5% of the N370S/others had low BMD (Table 2). These results are in concordance with the correlation between *GBA*1 genotypes and S-MRI score published elsewhere [16].

Significantly different proinflammatory profiles were observed in relation to GD genotype (Fig 5). N370S homozygous showed significantly high levels of MIP-1α and anti-inflammatory IL-10. Before ERT became available, splenectomy was frequently performed to control the adverse effects of an enlarged spleen in most affected patients. However, over time, an association between splenectomy and the exacerbation of GD-related bone disease has emerged [12]. For this reason, we decided to stratify the groups according to spleen status. We observed significant differences between splenectomized and non-splenectomized patients with regard to S-MRI scores. Eighty percent of non-splenectomized patients had a low normal score (Fig 1), which supported the clinical findings [12].

Genetic screening with respect to spleen status revealed three SNPs significantly associated with infiltration and bone lesions. In splenectomized patients, the polymorphism found in this study related with BMD protective effect *ESRI* c.453-397 T/T and *VDR* c.-83-25988 G/A heterozygous had a low-normal S-MRI score. Yamamoto et al.[35] described the *VDR* c.-83-25988 polymorphism as a functional binding site for the intestine-specific transcription factor Cdx-2 in the promoter region of the *VDR* gene. Subsequently, Arai et al.[36] described a G-to-A substitution at this Cdx-2 site that was found to modulate the intestine-specific transcription of the *VDR* gene. In addition, 100% of non-splenectomized patients with the *TNFRSF11B* c.9 C/C polymorphism exhibited low-normal S-MRI scores.

The *TNFRSF11B* gene encodes osteoprotegerin (OPG), a new member of the tumor necrosis factor receptor superfamily and a key regulator of bone remodeling [37]. OPG protects bone from excessive resorption by inhibiting the terminal stages of osteoclastogenesis [37], thereby suppressing mature osteoclast activation [38] and inducing osteoclast apoptosis [39]. This nucleotide substitution causes a change in the third amino acid in the signal peptide of OPG, from lysine to asparagine (p. K3N) [23]. The change of a basic lysine to asparagine (an uncharged polar amino acid) might influence the intracellular trafficking or export efficiency of the protein [40].

GD is associated with the release of several proinflammatory cytokines [10, 41]. We have observed high levels of IL-4, MIP-1α, MIP-1β, and TNFα, and lower levels of anti-inflammatory IL-10 and IL-13 in GD male and female patients compared with healthy control participants. The main chemotactic factors involved in the recruitment of mononuclear cells are monocyte chemotactic protein-1 (MCP-1) and macrophage inflammatory proteins MIP-1α and MIP-1β, which have been shown to increase bone resorption by osteoclasts in multiple myeloma [8]. Macrophage inflammatory proteins are also elevated in GD patients with bone disease [9, 10]. The IL-4, IL-13, and TNFα profiles have been reproduced in an *in vitro* GD model induced by Conduritol B epoxide (CBE) in peripheral blood mononuclear cells (PBMCs) [42]. The addition of CBE plus lipopolysaccharides increased TNFα secretion, and a tendency toward a reduction in the secretion of the T-cell derived cytokines IL-10 and IL-13 was observed. However, the exposure of PBMCs to CBE induced increased production of IL-4, which might reflect the differentiation of macrophages into alternative phenotypes [42]. Kacher et al.[43] showed that macrophages from a mouse model of GD, the L444P mouse, release significantly less IL-10 than their untreated counterparts. The reduced IL-10 secretion observed in GD mouse macrophages may be relevant to explain the increase in inflammation that is often observed in GD [43]. Surprisingly, we did not find significantly different plasma cytokine profiles according to BMD or S-MRI score, probably due to high inter-individual variability. ERT resulted in low cytokine profiles in plasma samples (Figs 2 and 3). ERT has been shown to produce short-term improvements in visceral and haematological complications and biomarkers in GD1 [44]. This improvement, due to glucosylceramide reduction, is reflected in the reduction of proinflammatory cytokines. The response of bone disease to ERT is much slower in adult patients with GD [45]; it is possible that early action of ERT on the Gaucher cells, avoiding the alteration of the immune system, achieves better results in the treatment of bone disease. BMD has been assessed before starting ERT. However, it may be important to analyze the degree of bone involvement in relationship with the timing of ERT initiation. It could be possible that patients with more severe bone disease are those who initiated ERT later; therefore they would have longer severe manifestations of GD. We analyzed the impact of splenectomy on proinflammatory profiles, and found that the median concentrations of all cytokines in untreated GD1 plasma samples are higher among non-splenectomized than splenectomized patients. These results are consistent with other published results that correlate spleen volume with disease plasma markers such as chitotriosidase or chemokine PARC/CCL18 [46]. Splenectomized GD patients had higher S-MRI scores, indicative of more severe bone disease, than non-splenectomized patients (Fig 1); this information supports clinical findings [12]. Splenectomy increases the number of Gaucher cells infiltrated into bone marrow, boosting the immunological alteration in the bone microenviroment caused partially by Gaucher mesenchymal stromal cells (MSCs].Campeau et al [47] find that Gaucher MSCs display an altered cytokine secretome that may be important in the bone and immune alterations. Concerning ERT therapy and splenectomy, we observed that cytokine levels were more sensitive to therapy in non-splenectomized patients. Among non-splenectomized patients, we observed significantly lower levels of MIP-1α, MIP-1β, and TNFα in ERT-treated versus untreated GD1 patients (Fig 4). The lack of difference in these cytokines between ERT-treated and untreated groups of splenectomized patients suggests that the immunological burden is lower in circulating blood and therefore greater in the bone microenviroment than non-splenectomized patients. It is unlikely that splenectomized patients have different susceptibility for bone involvement than those not spelenectomized and probably the fact that splenectomized patients have greater chronic inflammatory burden is independent of genetic predisposition. In this case the genetic background could be acting as an inmunomodulatory modifier, minimizing infiltration and bone lesions.

We observed significant differences between some cytokine concentrations and different SNP genotypes located at the *ESR1*, *VDR*, *BDNF*, *RUNX*, and *OPN* genes (Table D in S1 File). Therefore, genetic background not only directly influences bone metabolism, but can also act as a genetic modifier of inflammation. The retrospective analysis of proinflammatory cytokines in patients’ plasma samples is a limitation of this study. More basic research studies in cell or animal models will be required to better understand bone disease and develop new therapies against the immunomodulation of Gaucher cells.

## Conclusion

Our data suggest that in patients with GD, genetic background is fundamental to regulate the proinflammatory effect on bone disease development. Patients with GD1 are more susceptible to developing bone disease owing to the coexistence of genetic variants that increase the risk of bone involvement, including *GBA* genotypes. The proinflammatory cytokine profile changes according to the degree of response to ERT. Early treatment likely avoids disruption of the immune system, and consequently the development of BD, by reducing chronic inflammation produced by Gaucher cells in the bone marrow. This study supports the hypothesis that in Gaucher disease it is important to reverse the chronic alteration of the immune system by an early and personalized treatment avoiding the splenectomy in order to avert the cascade of the subsequent cytokines, reducing the number and intensity of bone complications.

## Supporting Information

S1 File Fig A, BUA (db/MHz) values among normal, osteopenic, and osteoporotic GD1 patients. **Fig B,** Comparison of plasma cytokine profiles in non-splenectomized versus splenectomized patients. A) Treatment-naïve GD1 patients; B) ERT-treated GD1 patients. **Table A,** GD1 Minor Allele Frequency (MAF), 1000 Genomes European Population MAF and Hardy-Weinberg Equilibrium (HWE). **Table B,** SNPs, Genotype, and BMD Association. **Table C,** SNPs, Genotype, and S-MRI Association Stratified by Spleen Status. **Table D,** Significant Differences in Cytokine Levels (pg/ml) Among SNP Genotypes. **Table E,** Primers and Probes. **Text A,** Genotyping.
(DOCX)
