# Supplementary material for: The Influence of Genetic Variability and Proinflammatory Status on the Development of Bone Disease in Patients with Gaucher Disease
Source: PLoS One. 2015 May 15;10(5):e0126153. doi: 10.1371/journal.pone.0126153 (PMC4433334; doi:10.1371/journal.pone.0126153)

**Table A.** GD1 Minor Allele Frequency (MAF), 1000 Genomes European Population MAF and Hardy-Weinberg Equilibrium (HWE)

| Gene | Polymorphism^a^ | rs Identification |  | GD1 MAF | HWE | 1000 Genomes EUR |  |
| --- | --- | --- | --- | --- | --- | --- | --- |
|  |  |  | Allele | Frequency | *p* | Frequency | *p* |
| *HSD11B1* | c.-48-7324A>T | rs4844880 | A | 0.12 | 0.33 | 0.16 | **0.001** |
| *TNFRSF11B* | c.9C>G | rs2073618 | C | 0.48 | 0.65 | 0.478 | 0.923 |
|  | c.-223C>T | rs2073617 | G | 0.4 | 0.73 | 0.478 | 0.086 |
| *RUNX2* | c.59-1041T>C | rs7771980 | C | 0.12 | **0.04** | 0.096 | 0.448 |
| *TGFB1* | c.142+76A>G | rs1800469 | A | 0.35 | 0.65 | 0.307 | 0.072 |
| *ESR1* | (TA)_n_ | rs3138774 | H | 0.47 | 0.88 | Unknown |  |
|  | c.453-397T>C | rs2234693 | C | 0.52 | 0.81 | 0.402 | **0.006** |
|  | c.453-351A>G | rs9340799 | G | 0.37 | 0.26 | 0.29 | **0.039** |
| *TNFSF11* | c.219+2528T>C | rs9594782 | C | 0.07 | 0.36 | 0.034 | **0.023** |
| *BDNF* | c.196G>A | rs6265 | T | 0.28 | **0.05** | 0.203 | **0.029** |
| *VDR* | c.-83-25988G>A | rs11568820 | T | 0.2 | 0.8 | 0.228 | 0.276 |
|  | c.152T>A | rs2228570 | A | 0.38 | 0.06 | 0.397 | 0.674 |
|  | c.1024+283G>A | rs1544410 | T | 0.4 | 0.57 | 0.404 | 0.979 |
|  | c.1025-49G>T | rs17879735 | A | 0.44 | 0.42 | 0.554 | **0.016** |
|  | c.1056T>C | rs731236 | G | 0.4 | 0.28 | 0.401 | 0.957 |
|  | c.-83-23269A>G | rs4516035 | C | 0.49 | **0.003** | 0.431 | 0.234 |
|  | c.-83-23777G>C | rs7139166 | G | 0.44 | 0.1 | 0.431 | 0.829 |
| *COL1A1* | c.-2116T>G | rs1107946 | A | 0.23 | **0.05** | 0.135 | **0.003** |
|  | c.-1782delT | rs11327935 | - | 0.2 | 0.41 | 0.199 | 0.961 |
|  | c.104-441G>T | rs1800012 | A | 0.2 | 0.29 | 0.202 | 0.817 |
| *IL6R* | c.-208G>A | rs4845617 | G | 0.45 | 0.78 | 0.392 | **0.001** |
|  | c.1073A>C | rs8192284 | C | 0.4 | 0.34 | 0.36 | 0.231 |
| *OPN* | c.-260_-259insG | rs11439060 | insG | 0.3 | 0.89 | 0.332 | 0.470 |
| *CLCN7* | c.1180G>A | rs12926089 | T | 0.15 | 0.44 | 0.104 | 0.086 |
| *VEGF* | c.-94C>G | rs2010963 | C | 0.32 | 0.17 | 0.297 | 0.542 |
| *BMP4* | c.455T>C | rs17563 | C | 0.45 | 0.86 | 0.437 | **0.013** |

^a^HGVS nomenclature

**Table B.** SNPs, Genotype, and BMD Association

| ***ESR1*** |  | **Genotype frequency, n (%)** |  | ***p*** |
| --- | --- | --- | --- | --- |
| **c.453-397T>C^a^** | **T/T** | **C/T** | **C/C** | **0.038** |
| Normal | 4 (48.3) | 8 (44.4) | 2 (40) |  |
| Osteopenia | 2 (33.3) | 8 (44.4) | 0 (0) |  |
| Osteoporosis | 0 (0) | 2 (11.1) | 3 (60) |  |
| **VDR** |  | **Genotype frequency, n (%)** |  | ***p*** |
| **c.1024+283G>A^b^** | **A/A** | **G/A** | **G/G** | **0.039** |
| Normal | 7 (87.5) | 9 (42.9) | 12 (66.7) |  |
| Osteopenia | 1 (12.5) | 9 (42.9) | 6 (33.3) |  |
| Osteoporosis | 0 (0) | 4 (19) | 0 (0) |  |

**a** Evaluated by DEXA; **b** Evaluated by ultrasound densitometry.

**Table C.** SNPs, Genotype, and S-MRI Association Stratified by Spleen Status

| **Splenectomized Patients, n=21** | | |  |  |
| --- | --- | --- | --- | --- |
| ***ESR1*** |  | **Genotype frequency, n (%)** |  | ***p*** |
| **c.453-397T>C** | **C/C** | **C/T** | **T/T** | **0.047** |
| Low-normal | 1 (33.3) | 2 (14.3) | 3 (75) |  |
| Mild | 2 (66.7) | 4 (28.6) | 0 (0) |  |
| Severe | 0 (0) | 8 (57.1) | 1 (25) |  |
| **VDR** |  | **Genotype frequency, n (%)** |  | ***p*** |
| **c.-83-25988G>A** | **A/A** | **A/G** | **G/G** | **0.045** |
| Low-Normal | 0 (0) | 3 (50) | 4 (33.3) |  |
| Mild | 0 (0) | 3 (50) | 4 (33.3) |  |
| Severe | 3 (100) | 0 (0) | 4 (33.3) |  |
| **Non-splenectomized Patients, n=62** | | |  |  |
| ***TNFRSF11B*** |  | **Genotype frequency, n (%)** |  | ***p*** |
| **c.9C>G** | **C/C** | **C/G** | **G/G** | **0.040** |
| Low-Normal | 15 (100) | 27 (84.4) | 9 (60) |  |
| Mild | 0 (0) | 2 (6.3) | 4 (26.7) |  |
| Severe | 0 (0) | 3 (9.4) | 2 (13.3) |  |

**Table D.** Significant Differences in Cytokine Levels (pg/ml) Among SNP Genotypes

| **Gene** | **SNP** | **Genotype cytokine median values (range)** | | | ***p*^a^** |
| --- | --- | --- | --- | --- | --- |
| ***ESR1*** | **n; (TA)n** | **18 H/H** | **45 H/L** | **25L/L** |  |
|  | TNFα | 13.90 (6.5-29.43) | 15.95 (3.6-98.15) | 12.26 (3.5-41.45) | 0.036 |
| ***VDR*** | **n; c.152T>A** | **31 C/C** | **48 T/C** | **9 T/T** |  |
|  | TNFα | 10.72 (3.5-34.75) | 17.73 (3.9-98.15) | 9.44 (4.15-13.01) | 0.012 |
|  | **c.1024+283G>A** | **17 A/A** | **39 A/G** | **32 G/G** |  |
|  | IL10 | 3.2 (3.2-18.05) | 3.2 (3.2-35.60) | 3.2 (3.2-70.47) | 0.039 |
|  | **c.-83-25988G>A** | **7 A/A** | **30 G/A** | **51 G/G** |  |
|  | IL13 | 3.2 (3.2-3.2) | 3.2 (3.2-240.62) | 3.2 (3.2-71.59) | 0.049 |
|  | TNFα | 13.1 (9.62-23.12) | 14.05 (4.15-34.27) | 14.81 (3.5-98.15) | 0.004 |
| ***BDNF*** | **c.196G>A** | **13 A/A** | **27 G/A** | **48 G/G** |  |
|  | IL10 | 3.2 (3.2-36.68) | 3.2 (3.2-70.47) | 3.2 (3.2-52) | 0.021 |
| ***RUNX2*** | **c.59-1041T>C** | **7 C/C** | **16 C/T** | **65 T/T** |  |
|  | IL10 | 3.2 (-) | 3.2 (3.2-70.47) | 3.2 (3.2-52) | 0.012 |
|  | IL6 | 3.2(-) | 7.23 (3.2-386.01) | 3.2 (3.2-56.90) | 0.001 |
|  | MIP-1α | 9.8 (3.2-8.53) | 81.98 (3.2-368.39) | (3.2-175.93) | 0.006 |
| ***OPN*** | **c.-260_-259insG** | **43 G/G** | **34 G/GG** | **11 GG/GG** |  |
|  | IL10 | 3.2 (3.2-30.88) | 3.2 (3.2-52) | 3.2 (3.2-70.47) | 0.036 |
|  | IL7 | 3.2 (3.2-100.34) | 3.2 (3.2-174.85) | 5.47 (3.2-345) | 0.043 |

**a** Kruskall-Wallis non-parametric test

**Table E.** Primers and Probes

| GEN | **SNP** | **rs#** | **SEQUENCE 5'→3'** | **PRODUCT (bp)** | **Technique** | |  |  |  |
| --- | --- | --- | --- | --- | --- | --- | --- | --- | --- |
| OPN | c.-260_-259insG | rs11439060 | GAT GCT GAA TGC CCA TCC CGT AAA T |  |  |  |  |  |  |
|  |  |  | CTG AGA CGA GTC TGG TCC CGA CGA T | 301 | SEQ |  |  |  |  |
| COL1A1 | c.-1782delT | rs 11327935 | CCC TGC CTC TCT GGA AAC TCT AT |  |  |  |  |  |  |
|  |  |  | CTC CCC CAA ACC ATC CAA GAT TC | 235 | SEQ |  |  |  |  |
|  | c.-2116T>G | rs1107946 | TGC CCT AGA CCA CCA CTC TAA AT |  |  |  |  |  |  |
|  |  |  | CAG CTG GTT TTG TGC AAC GA | 219 | RFLP | Alw26I |  |  |  |
|  | c.104-441G>T | rs1800012 | GGG CTT TTA AGA TGT CTA GGT GC |  |  |  |  |  |  |
|  |  |  | GGG AGT GGC TTG CGT GGT AGA GA | 218 | RFLP | Van91I |  |  |  |
| HSD11B1 | c.-48-7324A>T | rs4844880 | AGA GCA TTT CAA TGG TGT TT |  |  |  |  |  |  |
|  |  |  | TGG GTG GAG GAA TCA TCT A | 90 | SNaPshot |  |  |  |  |
| TNFRSF11B (OPG) | c.9C>G | rs2073618 | AGG TTT CCG GGG ACC AC |  |  |  |  |  |  |
|  |  |  | GGG AGG TTG GGA GAC CAG | 121 | SNaPshot |  |  |  |  |
|  | c.-223C>T | rs2073617 | AGA CAC CAC CGC CCC ACC CCT CAC G |  |  |  |  |  |  |
|  |  |  | TCG GCT GGC CCA GGG ACT TAC CAC G | 347 | RFLP | KspAI |  |  |  |
| RUNX2 | c.59-1041T>C | rs7771980 | AAA TCT TTT TCC CTT GGG AGT |  |  |  |  |  |  |
|  |  |  | AAG AGA TTG AAG CCG AGA CA | 182 | SNaPshot |  |  |  |  |
| TGFB1 | c.-1347T>C | rs1800469 | TTA GCC ACA TGG GAG GTG CTC AGT AAA G |  |  |  |  |  |  |
|  |  |  | AAA GAG GAC CAG GCG GAG AAG GCT TAA T | 251 | SNaPshot |  |  |  |  |
| ESR1 | (TA)_n_ | rs3138774 | TCC AAG ATT ATA GAC GCA TGA TA |  |  |  |  |  |  |
|  |  |  | TAC AAC TCG ATC TTC TCG GTT CA | 218 | CAP. ELEC | |  |  |  |
|  | c.453-397T>C | rs2234693 | ATC CAG GGT TAT GTG GCA ATG ACG TAA |  |  |  |  |  |  |
|  |  |  | AGG GGA AAT TGT TTA TTG CAA ACT TGT | 317 | RFLP | PvuII |  |  |  |
|  | c.453-351A>G | rs9340799 | CTG AGT TCC AAA TGT CCC A |  |  |  |  |  |  |
|  |  |  | CCT TGA GGG GAA ATT GTT TAT | 243 | SNaPshot |  |  |  |  |
| TNFSF11 (RANKL) | c.219+2528T>C | rs9594782 | GAG AAT AGT CTC CAT TTG ACT TGC |  |  |  |  |  |  |
|  |  |  | AAA CTG CTT TTC CTT GAG GAC T | 273 | SNaPshot |  |  |  |  |
| BDNF | c.196G>A | rs6265 | CGT CCA GGG TGA TGC TC |  |  |  |  |  |  |
|  |  |  | TGT AAC CCA TGG GAT TGC | 303 | SNaPshot |  |  |  |  |
| VDR | c.-83-25988G>A | rs11568820 | AGA GAA AGG AAG AAA AAG GAA T |  |  |  |  |  |  |
|  |  |  | CAT TTA TAT TTC CTA ACC CAA GTT | 333 | SNaPshot |  |  |  |  |
|  | c.152T>A | rs2228570 | TGG CAC TGA CTC TGG CTC TGA CCG T |  |  |  |  |  |  |
|  |  |  | CTT TGC AGC CTT CAC AGG TCA TAG CAT T | 185 | RFLP | BseGI |  |  |  |
|  | c.1024+283G>A | rs1544410 | GCA GAG CCC CTG TGG TGT GTG GAC G |  |  |  |  |  |  |
|  |  |  | CAT TGC CTC CAA AAT CAA TCA GGT A | 285 | RFLP | Hin6I |  |  |  |
|  | c.1025-49G>T | rs17879735 | CCT AGG TCT GGA TCC TAA ATG CAC G |  |  |  |  |  |  |
|  |  |  | CTC CTC ATT GAG GCT GCG CAG GTC G | 366 | RFLP | ApaI |  |  |  |
|  | c.1056T>C | rs731236 | CCT AGG TCT GGA TCC TAA ATG CAC G |  |  |  |  |  |  |
|  |  |  | CTC CTC ATT GAG GCT GCG CAG GTC G | 366 | RFLP | TaqI |  |  |  |
|  | c.-83-23269A>G | rs4516035 | TCC TCT GTA AGA GGC GAA TAG CGA T |  |  |  |  |  |  |
|  |  |  | TGA CTG TGT GAT AGG CAC CGC TCT A | 229 | RFLP | Eco32I |  |  |  |
|  | c.-83-23777G>C | rs7139166 | AAA AGT GAC TTC TGA GTG TTC AAC C |  |  |  |  |  |  |
|  |  |  | AGA CAG AAG AGA GGC ATA GCG TTT G | 283 | RFLP | Van91I |  |  |  |
| IL6R | c.-208G>A | rs4845617 | GCT GAT ACG CCC TTT TCT CAT CAA C |  |  |  |  |  |  |
|  |  |  | CCC GGC TCT CTA CAC ACA CTG CGA G | 350 | RFLP | BcnI |  |  |  |
|  | c.1073A>C | rs8192284 | AGC TTG TCA AAT GGC CTG TTG GTT G |  |  |  |  |  |  |
|  |  |  | GGC AAT GCA GAG GAG CGT TCC GAA G | 261 | RFLP | HindIII |  |  |  |
| CLCN7 | c.1180G>A | rs129266089 | CCA CGG GCC CAG TGT CCT CCA TCA G |  |  |  |  |  |  |
|  |  |  | TGG CAA TCC CGC GAC GAG TAG ATC A | 206 | RFLP | Hin1 II (Nla III) | |  |  |
| VEGF | c.-94C>G | rs2010963 | GCT CCA GAG AGA AGT CGA GG |  |  |  |  |  |  |
|  |  |  | TGC GGG ATC CCA AGG | 151 | SNaPshot |  |  |  |  |
| BMP4 | c.455T>C | rs17563 | TAA CCT CAG CAG CAT CCC TGA GAA CGA CG |  |  |  |  |  |  |
|  |  |  | GGG CTC ACA TCA AAA GTT TCC CAC CGT GTC | 232 | RFLP | Bsh1236I |  |  |  |
| SNaPshot Probes |  |  |  |  |  |  |  |  |  |
| HSD11B1 | c.-48-7324A>T | rs4844880 | GAGCATTTCAATGGTGTTTTTATTC |  |  |  |  |  |  |
| TNFRSF11B (OPG) | c.9C>G | rs2073618 | AAAAAAAAAAAAAAGGTTTCCGGGGACCACAATGAACAA |  |  |  |  |  |  |
| VEGF | c.-94C>G | rs2010963 | AAAAAAAAAAAAAAAAAAAAAACGCGCGGGCGTGCGAGCAGCGAAAG | |  |  |  |  |  |
| RUNX2 | c.59-1041T>C | rs7771980 | AAAAAAAAAAAAAAAAAAAAAAAAAAAAAATCAGCACAGAGGCTCAAGAATTTTC | | |  |  |  |  |
| TGFb1 | c.-1347T>C | rs1800469 | AAAAAAAAAAAAAAAAAAAAAAAAAAAAAAAAAAAAAATGTCTGCCTCCTGACCCTTCCATCC | | |  |  |  |  |
| ESRI | c.453-351A>G | rs9340799 | AAAAAAAAAAAAAAAAAAAAAAAAAAAAAAAAAAAAAAAAAAAAAATAGAGACCAATGCTCATCCCAACTC | | | |  |  |  |
| RANKL | c.-1347T>C | rs1800469 | AAAAAAAAAAAAAAAAAAAAAAAAAAAAAAAAAAAAAAAAAAAAAAAAAAAAAATGGAACTTGGTACCAAATAAATCAG | | | | |  |  |
| BDNF | c.196G>A | rs6265 | AAAAAAAAAAAAAAAAAAAAAAAAAAAAAAAAAAAAAAAAAAAAAAAAAAAAAAAAAAAAAAATCATTGGCTGACACTTTCGAACAC | | | | | |  |
| VDR | c.-83-25988G>A | rs11568820 | AAAAAAAAAAAAAAAAAAAAAAAAAAAAAAAAAAAAAAAAAAAAAAAAAAAAAAAAAAAAAAAAAAAAAAATATTCCTGAGTAAACTAGGTCACA | | | | | | |

**Text A.**Genotyping

**1-Analysis of SNPs using restriction fragment length polymorphism analysis**

We used restriction fragment length polymorphism (RFLP) analysis to assess the following polymorphisms: c.104-441G>T and c.-2116T>G SNPs of *COL1A1*; c.152T>A, c.1024+283G>A, c.1025-49G>T, c.1056T>C, c.-83-23269A>G, and c.-83-23777G>C of *VDR*; c.453-397T>C of *ESR1*; c.-208G>A and c.1073A>C of *IL6R*; c.1180G>A of *CLCN7*; c.455T>C of *BMP4*; and c.-223C>T of *TNFRSF11B*. Briefly, fragments were generated in a PCR volume of 10 μL, which contained 100 ng of genomic DNA by optimized protocols. Two microliters of each PCR product were digested with 0.2 μL of restriction enzyme (Table E), 0.8 μL Enzyme Buffer, and 5 μL ultrapure DNAase/RNase-free distilled water; reactions were incubated for 240 min at 37ºC or 55ºC (BseG I) or 65ºC (Taq I). The digestion products were electrophoresed on a 3% agarose gel in 1× TAE buffer at 94V for 40 min. Ten percent of the samples were sequenced to check the results.

**2-Analysis of the microsatellite (TA)n repeat in the promoter region of ESR1**

This repeat polymorphism was examined using a 10-μL PCR volume that contained 100 ng of genomic DNA, using a forward primer labelled at the 5’ end with the FAM fluorophore (Table E). Five microliters of PCR product were diluted 1/20, and 0.8 μL of the dilution was mixed with 0.2 μL GenesScan™ 600 LIZ® dye Size Standard v2.0 (Applied Biosystems) and 9 μL Hi-Di™ formamide (Applied Biosystems); finally, the mixture was denatured at 90ºC for 2 min. Samples were resolved in an Applied Biosystems 3500XL Genetic Analyzer automated DNA sequencer (Applied Biosystems). GeneMapper® v4.1 Software (Applied Biosystems) was used to analyse the results. Simultaneously, a homozygous sample was cloned and sequenced to compare the number of repeats. The repeat alleles were clustered into two groups (L=9-17 repeats and H=18-26 repeats, as described elsewhere) [1].

**3-Analysis of SNPs using the Sanger sequencing method**

Sequencing was used to type the c.-1782delT of *COL1A1* and the c.-260_-259insG of *OPN.* PCR fragments were generated in a reaction volume of 10 μL that contained 100 ng of genomic DNA. Amplicons were purified through enzymatic methods using Illustra™ ExoProStar™ 1-Step (GE Healthcare), following the manufacturer’s recommendation. Bi-directional Sanger sequencing was conducted using the BigDye® Terminator v3.1 Cycle Sequencing Kit (Applied Biosystems, following manufacturer’s protocols) and an Applied Biosystems 3500XL Genetic Analyzer automated DNA sequencer (Applied Biosystems). Variant Reporter® v1.1 Software (Applied Biosystems) was used to analyse the results.

**4-Analysis of SNPs using SNaPshot**

The SNaPshot® method (Applied Biosystems) was used to type the following polymorphisms: c.453-351A>G of *ESR1*, c.-83-25988G>A of *VDR*, c.59-1041T>C of *RUNX2*, c.219+2528T>C of *TNFSF11*, c.142+76A>G of *TGFB1*; c.9C>G of *TNFRSF11B*, c.196G>A of *BDNF*, c.-48-7324A>T of *HSD11B1* and c.-94C>G of *VEGF*. The nine PCR fragments were generated through a multiplex PCR in a final volume of 10 μl that contained 50 ng of genomic DNA. All primer sequences used for PCR are available upon request. Primers and unincorporated dNTPs were removed by enzymatic methods using Illustra™ ExoProStar™ 1-Step (GE Healthcare), following the manufacturer’s recommendation. SNaPshot^®^ reactions were performed in 10 μl volumes using 3 μl of diluted (1/50) clean multiplex PCR product, 3 μl SNaPshot Multiplex Reaction Mix (Applied Biosystems), and each SNaPshot^®^ probe at a final concentration of 0.2 μM. The probes used for the SNaPshot^®^ reactions are described in the Supplemental Table 5. Unincorporated ddNTPs were removed by treatment with SAP (Affymetrix). Clean products were first mixed with Hi-Di™ Formamide (Applied Biosystems) and GeneScan™ 120 Liz^®^ size standard (Applied Biosystems), then denatured at 95ºC for 5 min and loaded onto a 3500XL Genetic Analyzer automated DNA sequencer (Applied Biosystems). GeneMapper^®^ v4.1 Software (Applied Biosystems) was used to analyse the data.

1. Bustamante, M., et al., *COL1A1, ESR1, VDR and TGFB1 polymorphisms and haplotypes in relation to BMD in Spanish postmenopausal women.* Osteoporos Int, 2007. **18**(2): p. 235-43.

**S1 Fig.** BUA (db/MHz) values among normal, osteopenic, and osteoporotic GD1 patients.


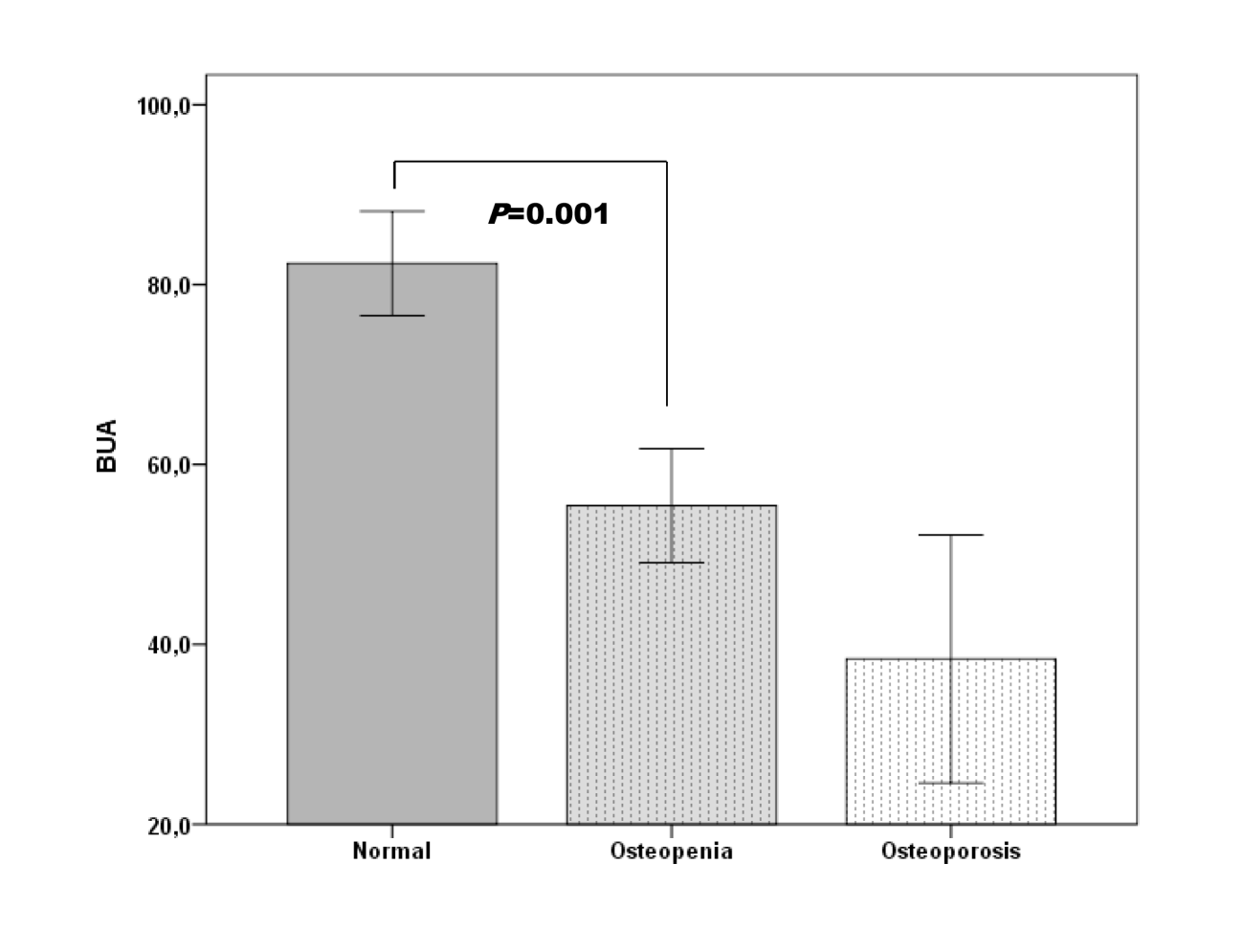


**S2 Fig.** Comparison of plasma cytokine profiles in non-splenectomized versus splenectomized patients. A) Treatment-naïve GD1 patients; B) ERT-treated GD1 patients.


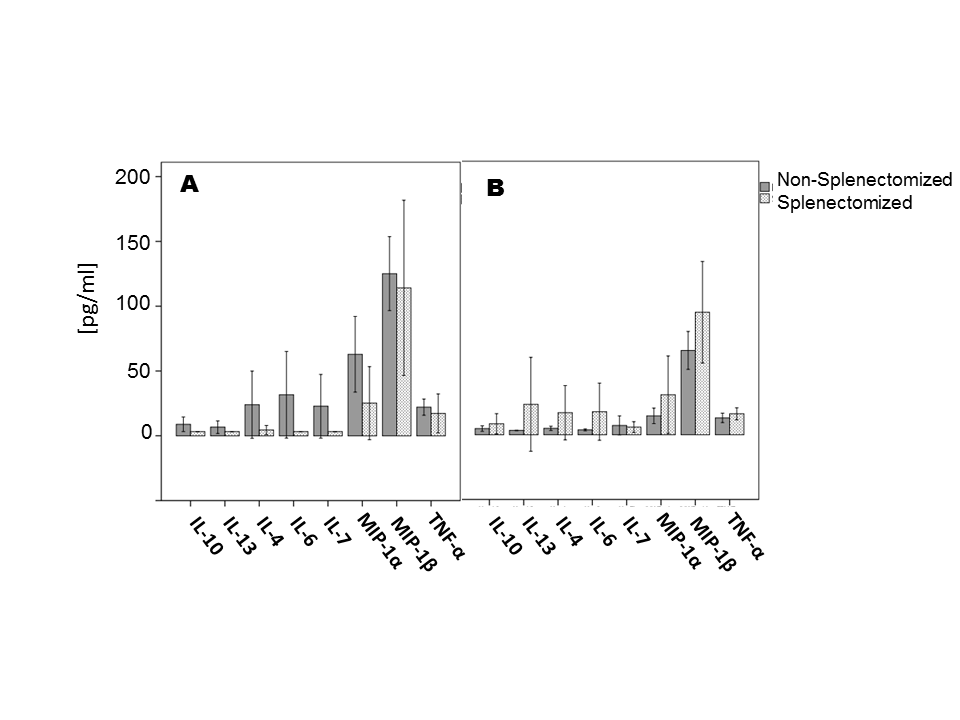

Supplement: S1 File — Fig B, Comparison of plasma cytokine profiles in non-splenectomized versus splenectomized patients. A) Treatment-naïve GD1 patients; B) ERT-treated GD1 patients. Table A, GD1 Minor Allele Frequency (MAF), 1000 Genomes European Population MAF and Hardy-Weinberg Equilibrium (HWE). Table B, SNPs, Genotype, and BMD Association. Table C, SNPs, Genotype, and S-MRI Association Stratified by Spleen Status. Table D, Significant Differences in Cytokine Levels (pg/ml) Among SNP Genotypes. Table E, Primers and Probes. Text A, Genotyping. (DOCX) [file pone.0126153.s001.docx]
